# Ceramic fragmentation after total hip arthroplasty: two case reports and literature review

**DOI:** 10.3389/fsurg.2024.1357301

**Published:** 2024-02-20

**Authors:** Tingyu Wu, Sijia Guo, Yaping Jiang, Weipeng Shi, Yingzhen Wang, Tao Li

**Affiliations:** ^1^ Department of Joint Surgery, The Affiliated Hospital of Qingdao University, Qingdao, China; ^2^ Department of Oral Implantology, The Affiliated Hospital of Qingdao University, Qingdao, China

**Keywords:** ceramic fragmentation, ceramic fracture, total hip arthroplasty, case report, literature review

## Abstract

**Background:**

Ceramic fragmentation is a rare but serious complication after total hip arthroplasty (THA). We reviewed the PubMed literature from 1990 to 2023 and found only 31 case reports of ceramic fragmentation after THA. Our case reports help to expand understanding of this rare complication. We shared our surgical experience and identified an ideal material for revision surgery, which can serve as a useful reference for other orthopedic surgeons to perform ceramic fragmentation revision surgery in the future. We also analyzed the possible causes, diagnosis, and treatment opinions of ceramic fragmentation.

**Case presentation:**

This study presents two cases of ceramic fragmentation after THA. One patient had ceramic head fragmentation 10 years after the primary THA, and one patient had ceramic liner fragmentation 5 years after the primary THA. Both patients presented with pain, and one patient also reported a clicking sound in the hip. The two patients described here had BMIs of 23.7 and 23.1, respectively. Both patients' ceramic fragmentation were due to aseptic loosening, not periprosthetic joint infections, as confirmed by negative microbiological cultures. Radiographic examinations of both patients revealed radio-opaque wear debris around the hip joint prostheses and we describe the surgical protocols and intraoperative findings in both cases in detail.

**Conclusion:**

Our cases and the literature suggest that ceramic fragmentation can occur at any time after THA. The most immediate symptoms are pain and noise, but some patients may be asymptomatic. Ceramic on polyethylene bearings is recommended for revision surgery whenever possible; metal bearings should be avoided.

## 1 Introduction

Ceramic fragmentation after total hip arthroplasty (THA) is a rare complication that was first reported by Israel et al. in 1989 as a potentially catastrophic complication requiring revision surgery (1). Although the incidence of ceramic fragmentation is low, ceramic fragments may induce metallosis or osteolysis (2).

Understanding the factors that contribute to ceramic fragmentation is helpful to prevent it after THA. The main causes of ceramic fragmentation after THA include a high body mass index (BMI) (3), direct or indirect trauma (4), surgical design flaws, such as the choice of bearing surface type (5), neck-cup impingement (6), small head size (4), surgical approach (7), and improper manipulation during the operation, such as misplacement of the acetabular liner (8) or incorrect ceramic insertion (9). Once ceramic fragmentation occurs, the most immediate symptom is sudden severe pain with functional impairment (9). However, ceramic fragmentation has been found incidentally in asymptomatic patients, suggesting that it may be underdiagnosed (10). Therefore, any indication for THA should prompt the orthopedic surgeon to be alert to the possibility of ceramic fragmentation.

In this report, we present two cases of ceramic fragmentation after THA: one following an accidental sprain and the other occurring suddenly with no obvious cause. Both patients had received primary THA in our hospital several years before. We describe their clinical and radiological features, treatment options, and outcomes to improve understanding of the disease, alert orthopedic surgeons to ceramic fragmentation after THA, reduce missed diagnosis and misdiagnosis, and provide an appropriate revision plan.

## 2 Case presentation

### 2.1 Case 1

**Figure 1:**
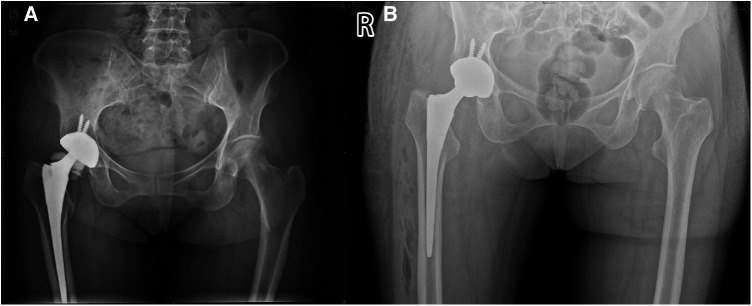
Pelvic and hip x-rays in case 1. (**A**) Preoperative anteroposterior radiographs of both hips. (**B**) Postoperative bedside DR anteroposterior radiographs of both hips.

**Figure 2:**
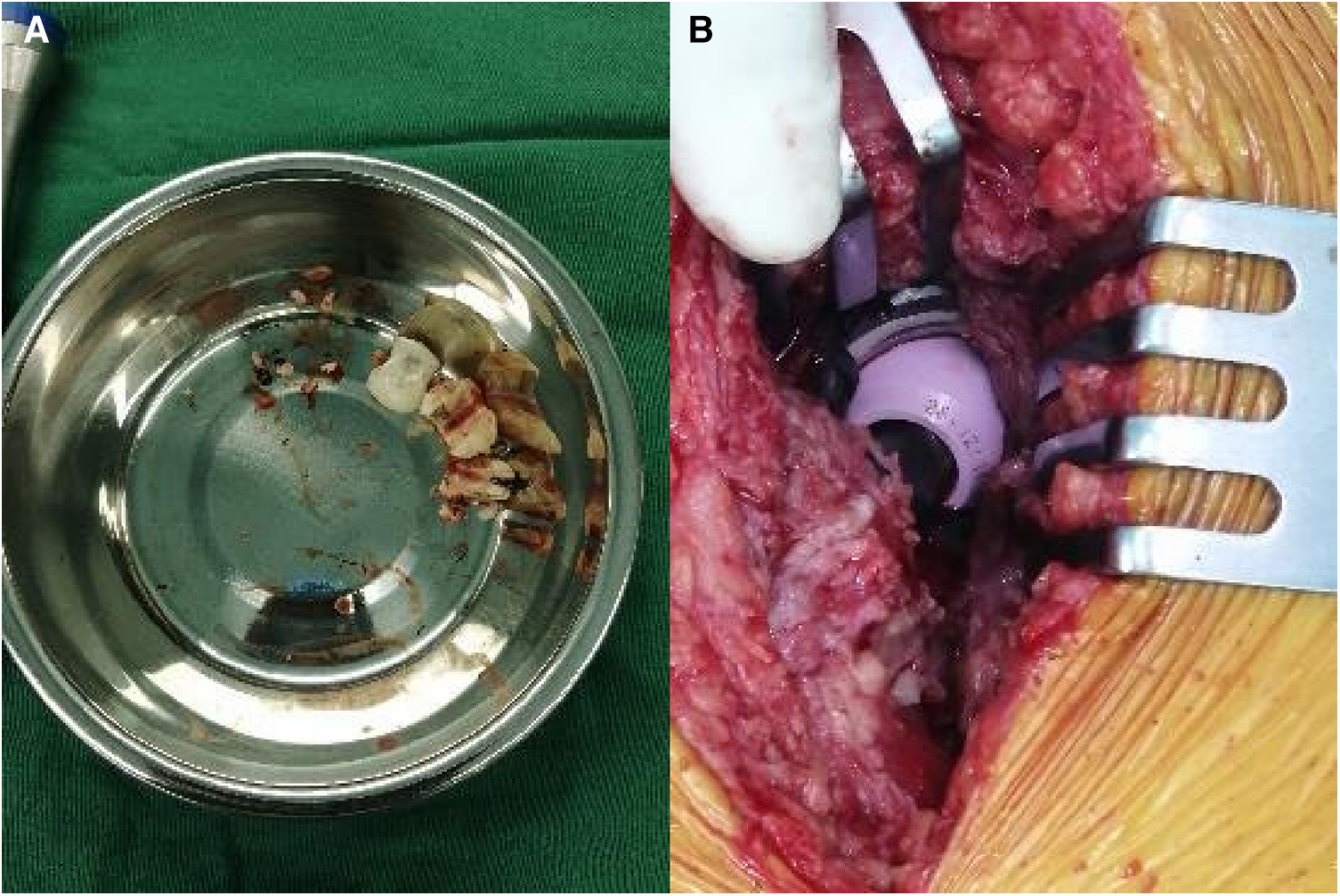
Revision surgery in case 1. (**A**) Ceramic fragments. (**B**) New 28 mm pink Aesculap ceramic head.

A 59-year-old woman, weighing 63 kg with a height of 163 cm and a BMI of 23.7 kg/m^2^, presented with a 10-day history of right hip discomfort with clicking sounds. She had undergone THA following a right femoral neck fracture 10 years earlier. Ten days ago, she inadvertently strained her hip while working on her farm, leading to the onset of discomfort and clicking in her hip joint. This incident was characterized by restricted movement but was not accompanied by redness, swelling, or abnormal skin temperature. The sudden onset of pain and a snapping sensation in her hip, following the strain, were particularly noteworthy. Upon presentation, she was in pain and limping, but there was no significant deformity or tenderness in the right hip joint. Hip radiography ruled out aseptic loosening or infection, and her blood parameters, including erythrocyte sedimentation rate (ESR) and C-reactive protein (CRP) were within the normal range. Plain radiograph revealed significant ceramic flaking (Figure 1A), prompting the recommendation for right hip prosthesis revision. During surgery, a fractured femoral head and surrounding bone spurs were discovered after a “T”-shaped incision in the joint Subsequently, we conducted proactive surgical debridement, cleared the proliferative tissues, and removed the fragmented femoral head prosthesis (Figure 2A). During the revision, it was found that only the ceramic head was broken, with no loosening detected, leading to the decision to replace it with a new pink Aesculap ceramic head. After testing the new 28 mm ceramic head, it was inserted, and the tightness was satisfactory (Figure 2B). The patient has recovered well after the surgery. Follow-up x-rays confirmed well-positioned acetabular and femoral prostheses with normal anteversion and abduction angles, as well as proper fit and stability, resulting in a satisfactory surgical outcome (Figure 1B). The pain was well controlled at the 2-week follow-up, and she returned to her normal activities six weeks after surgery.

### 2.2 Case 2

**Figure 3:**
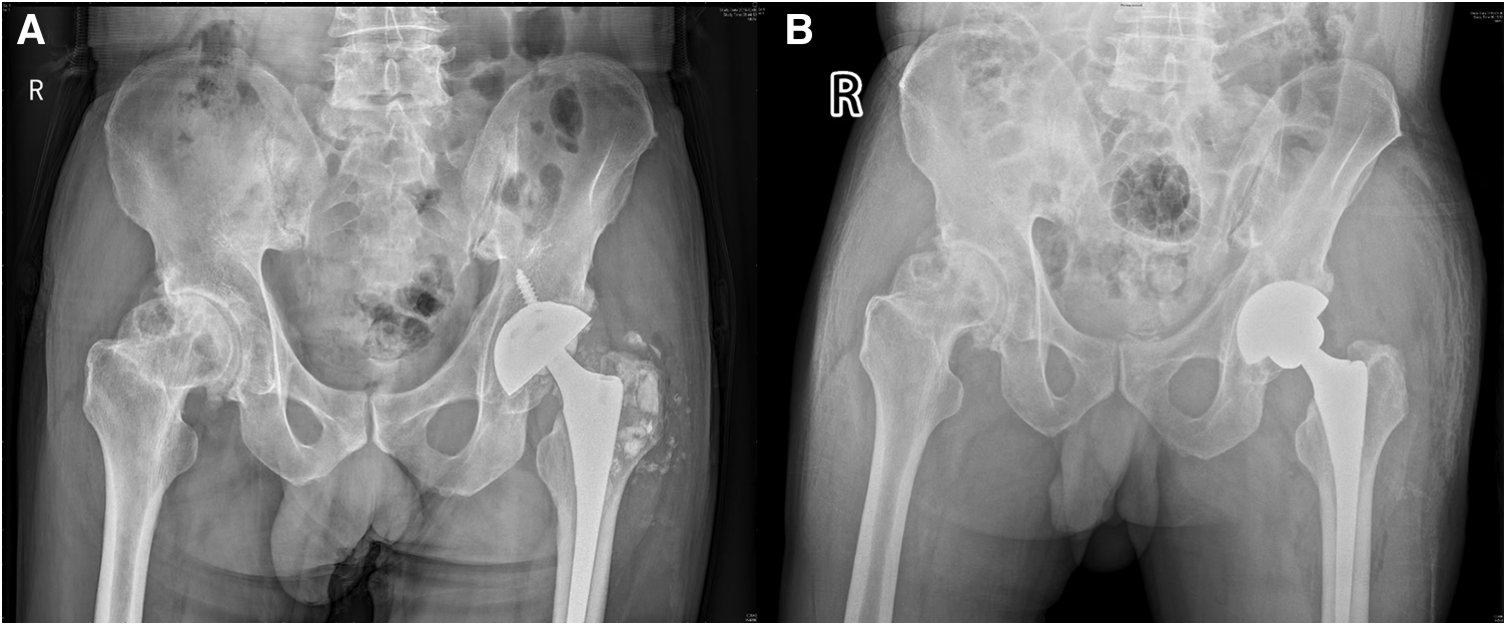
Pelvic and hip x-rays in case 2. (**A**) Preoperative anteroposterior radiographs of both hips. (**B**) Postoperative bedside DR anteroposterior radiographs of both hips.

**Figure 4:**
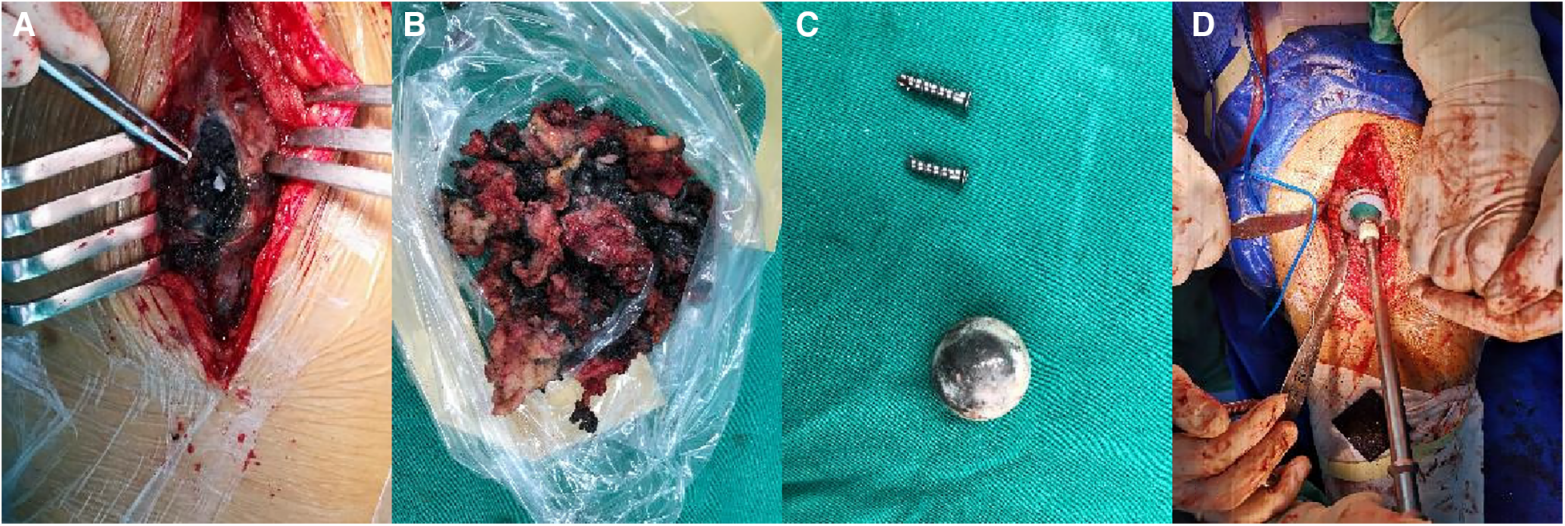
Revision surgery in case 2. (**A**) A large amount of black debris. (**B**) Tissue stained black by ceramic debris. (**C**) The removed screws and original yellow ceramic head. (**D**) Applying pressure to the polyethylene liner.

A 60-year-old man, weighing 66 kg, with a height of 169 cm and a BMI of 23.1 kg/m^2^, presented with left hip pain lasting for 1 month. He had previously undergone THA following a left femoral neck fracture 5 years earlier. The pain in his left hip developed suddenly one month ago, without any clear cause. It was described as dull and intermittent, worsening with excessive activity and weather changes, but there was no joint swelling or redness. Since his retirement, his lifestyle had become more sedentary, with only limited outdoor activities. Upon presentation, he arrived at the hospital on crutches; his lower limbs were of equal length, and he had tenderness in the left inguinal region and over the greater trochanter. Plain radiographs showed many small ceramic fragments flaking around the hip joint (Figure 3A). Therefore, he was scheduled for revision of the left hip prosthesis. After opening the joint capsule through a lateral incision and removing it, we discovered a fractured ceramic liner in the acetabulum. The presence of a significant amount of black wear debris and turbid fluid accumulation in the surrounding area suggested a more chronic process than initially apparent (Figure 4A). Despite the patient's history indicating symptoms for only one month, the extent of debris accumulation raises the possibility that the underlying issue may have been present for a longer duration. Subsequently, we completely excised the joint capsule and the tissues stained black due to the ceramic wear debris (Figure 4B). We then dislocated the joint, removed the femoral head prosthesis, and cleaned the surrounding synovial tissues. Then we removed the original screw and yellow ceramic head. There were many traces of metal on the surface of the ceramic head due to prolonged friction with the metal cup (Figure 4C). Although the acetabular cup was firmly fixed to the bone, we faced a challenge as the yellow ceramic liner had been discontinued. Consequently, we had to remove the original yellow ceramic head and opted to use a polyethylene liner, affixed to the original acetabulum with bone cement, while replacing the head with a zirconia one. To facilitate the penetration of bone cement, we removed the screws from the original acetabulum. If the acetabular prosthesis had been loose, it would have been possible to remove it for revision. The acetabular side surface was then roughened with an electric drill to ensure better adhesion. After placing the cement on the polyethylene liner, we maintained pressure for 10 min until the cement dried. This step was crucial for the successful adhesion of the cement. The operation was completed successfully. Postoperatively, we conducted microbial cultures on the intraoperative samples (tissue and synovial fluid), which resulted in negative findings. Although the patient showed a slight increase in ESR and C-reactive protein levels after surgery, it is likely due to trauma and stress.

After the revision surgery, the patient's left hip pain was significantly improved. Postoperative radiographs showed well-positioned acetabular and femoral prostheses with appropriate anteversion and abduction angles, and no signs of loosening. (Figure 3B). The patient had a satisfactory surgical outcome, was discharged on the fifth day, and received instructions on limb exercises, nutrition, and infection prevention. Pain resolved by the 3-week follow-up, and normal activities resumed six weeks post-surgery. The patient was readmitted two months later for congenital hip dysplasia and underwent right hip replacement surgery. Two months post-surgery, the patient had a good prognosis with no complications and satisfactory hip mobility. Long-term follow-ups showed excellent recovery, full hip motion without discomfort, improved daily function, and stable joint health without further interventions.

## 3 Discussion and conclusions

Ceramic fragmentation after THA is rare, with a 0.004% incidence of ceramic head fragmentation and 0.21% incidence of ceramic lining fragmentation (11). Our review of PubMed from 1990 to 2023 found 31 case reports of ceramic fragmentation after THA (Table 1). The male-to-female ratio was 2:1 and the average age was 52.6 (range 21–82) years. In these case reports, the interval from primary THA to revision ranged from a few days to several years, suggesting that ceramic fragmentation can occur at any time after THA. All patients who have undergone a THA should immediately have plain radiographs taken once they experience postoperative pain or an abnormal sound in the hip. When radio-opaque particles are seen around the prosthesis, orthopedic surgeons should be alert to the possibility of ceramic fragmentation and perform revision surgery immediately after diagnosis. If a ceramic fracture is suspected and cannot be identified on plain radiographs, over-penetration using image intensification should be considered to help identify a subtle ceramic liner fracture (30). Computed tomography can also play an important role in the diagnosis (13). Rapid diagnosis of ceramic fragmentation after surgery is particularly important, as a long delay may cause extensive bone damage with irreversible consequences (37). Generally, after the fracture of a ceramic femoral head, the resulting fragments tend to be larger due to the absence of further grinding interactions. On the other hand, after the fracture of a ceramic liner, as the ceramic head continues to interact with the corresponding component, it can further grind the liner, leading to the generation of smaller fragments. This was observed in both of our cases according to the preoperative x-ray images.

**Table 1:** Review of ceramic fragmentation after THA (1990–2023).

First author and reference	No. of cases	Age/sex	Precipitating factors	Preoperative symptoms	Time from primary THA to revision	Ceramic fracture type
Callaway (12)	4	21 F 62 M 52 M 55 F	Volleyball game; Climbed the stairs; Tripped; N	Crunching and pain; Slight discomfort; Intermittent groin pain; Snap and mild pain	5 M 6 M 8 M 9 M	Ceramic liner Ceramic head Ceramic head Ceramic head
Goretti (13)	2	58 M 82 M	N N	Noise Noise and pain	9 Y 11 Y	Ceramic liner Ceramic liner
McCarthy (14)	2	71 F 58 F	Sat and then stood; N	Pain; N	7 M 5 W	Ceramic liner Ceramic liner
Patetta (15)	1	45 M	Tripped and fell on his right hip	Severe pain in the right groin	4.5 Y	Fourth-generation ceramic head
Pawar (16)	1	29 M	N	Squeaking	30 M	Ceramic liner
Kern (17)	1	/	N	Pain	2 Y	Ceramic head
Stea (18)	1	60 M	Fell	A crunch with motion of the left hip	7 Y	Ceramic head
Xing (19)	1	50 M	Fell	Continuous crepitus in the right hip	6 Y	Ceramic head
Aytekin (20)	1	57 F	N	Pain	6 Y	Ceramic liner
Rankin (21)	1	52 M	Fell on his right hip	Sharp pain and a sensation of grinding in his right hip	8 M	Ceramic head
Otsuka (22)	1	48 M	N	Suddenly heard and felt a loud crack in his right hip	4 Y	Ceramic head
Heiner (23)	1	45 F	A bicycle accident	Moderately severe left hip pain	18 M	BIOLOX delta ceramic head
Valentini (24)	1	58 M	Direct trauma in skiing activity	Pain	6 Y	BIOLOX delta ceramic head
Pomeroy (25)	1	41 M	N	Clicking sound	10 D	BIOLOX delta ceramic head
Lee (26)	1	41 M	N	Pain	2 Y	Ceramic liner
Antoniac (27)	1	56 M	Road traffic accident	Pain	1 Y 3.5 Y	Ceramic liner
Artiaco (28)	1	72 M	N	Pain	3 Y	Ceramic liner
Topolovec (29)	1	50 M	N	Pain, crepitus noises, limited hip motion	11 Y	Ceramic liner
Shafafy (30)	1	82 M	N	Right groin pain	15 M	Ceramic liner
Gallo (31)	1	37 F	N	Squeaking noise accompanied each step	7 Y	Ceramic liner
Sodiwala (32)	2	39 M 46 M	Fell on right hip; N	Continuing crunching in the right hip; Sudden onset pain to the right hip	8 M 7 Y	Ceramic head Ceramic head
Bekler (33)	1	48 F	N	Pain, limitation of range of motion, sudden cracking sound	14 M	Ceramic liner
Dirvar (34)	1	50 M	Traumatic event	Pain, limb length shortening	5 Y	Ceramic liner
Hasegawa (35)	1	59 F	N	A crepitus deep in the right hip during motion	1 Y	Ceramic liner
Allain (36)	1	54 F	N	Sudden onset of pain in the hip	5 Y	Ceramic head

M, male; F, female; N, no; Y, years; M, months; D, days.

The causes of ceramic fragmentation are complex and multifactorial, encompassing various factors such as a high BMI, strenuous activity, direct or indirect trauma, and errors during the primary THA procedure. Although the BMI values of the patients in our two cases were within the normal range, it should be noted that high BMI, profession, and level of daily activities have been identified as potential risk factors for ceramic fragmentation (4, 38).

Additionally, factors related to the ceramic head itself, such as diameter, material, and impaction, also play a significant role in head fragmentation (39). In THA, it is advisable to avoid the use of short-neck ceramic femoral heads as they may carry a higher risk of fracture (40, 41). The design of short-neck ceramic femoral heads may make them more susceptible to fracture under specific stresses. This design characteristic could increase the stress concentration on the ceramic material, making it more vulnerable to damage. However, it is important to note that the specific choice of components should be based on individual patient's factors and bone anatomy. Short-neck ceramic femoral heads might be a suitable choice for certain patients, while they may not be appropriate for others. This includes factors such as a patient's bone quality, bone density, bone structure, age, activity level, and other potential risk factors. Therefore, during THA, it is essential to consider each patient's unique situation, carefully assess their bone condition and risk factors, and choose the most suitable implant components under the guidance of a healthcare professional.

To address the issue of ceramic fracture, the BIOLOX Delta ceramic femoral head was introduced and showed promise in reducing the incidence of fractures during early follow-up (42). However, despite these advancements, fractures of the BIOLOX Delta ceramic femoral head have still been reported. In a case report by Rankin et al. (21) in 2019, a fourth-generation BIOLOX Delta ceramic femoral head fracture was documented in a patient who experienced severe pain after swimming. The occurrence of ceramic fractures is still closely related to the surgeon's surgical technique. Improper impaction of the femoral head prosthesis during surgery has also been identified as a potential risk factor for ceramic head fracture (43). Although the use of fourth-generation ceramics significantly improves the material's resilience, making it less prone to brittle fracture, orthopedic surgeons still need to pay attention to obtaining proper head seating on a clean taper during femoral head assembly as a prerequisite to reduce the risk of any damage occurring at the head-taper junction (44). The surgeon's clinical experience and expertise play a crucial role in ensuring the success of the surgery and the safety of the patient.

Regarding ceramic liner fragmentation, factors such as size, material, and placement have been implicated as contributors to this issue. Ceramic liner fractures can be classified into two types: central fractures involving the entire liner and chip fractures at the peripheral rim (39). The occurrence of central fractures is often associated with incorrect installation of the ceramic liner (42). On the other hand, ceramic edge fractures are believed to result from an impact between the inner edge of the ceramic liner and the stem neck, but the exact mechanism remains unclear (45). When selecting the femoral stem, surgeons should be cautious and avoid using a large-diameter or thick-stemmed femoral component whenever possible. This is because such thick-stemmed femoral components can create a high risk of impingement with the ceramic liner, leading to ceramic liner fractures (6).

Ceramic liner fractures can occur due to impingement, which happens when the femoral stem's neck impinges against the inner edge of the ceramic liner during certain movements. The impact can cause stress concentrations on the ceramic liner and may result in fractures or chipping of the ceramic material. To minimize the risk of impingement-related ceramic fractures, surgeons should carefully assess the patient's anatomy and choose an appropriately sized femoral stem. Using a femoral component with a suitable neck length and diameter can help ensure sufficient clearance between the femoral stem and the ceramic liner, reducing the likelihood of impingement during joint movement. It is crucial for surgeons to consider the specific patient's anatomy, biomechanics, and implant compatibility when selecting the femoral stem and other components during total hip arthroplasty. A thorough preoperative evaluation and careful surgical planning are essential to achieve optimal outcomes and minimize potential complications, including ceramic liner fractures.

While we found that some patients had a history of direct or indirect trauma, the majority of patients had no obvious predisposing factors for ceramic fragmentation, and a few were asymptomatic when it occurred (summarized in Table 1). Our second patient had experienced sudden-onset pain without an obvious trigger or trauma. Lucchini et al. (44) demonstrated that ceramic head fracture can occur even without complications or significant previous trauma. Slight micromotion can lead to interface damage, leading to the loosening of the ceramic head and ultimately resulting in ceramic femoral head fracture.

Revision surgery should be performed immediately in patients with ceramic fragmentation to reduce the trauma to the surrounding tissue caused by the ceramic. It is important to be sure to remove all ceramic fragments, as this will increase the longevity of the new bearing (46). During revision surgery, the femoral head should be made of the same or stiffer material to prevent catastrophic wear and tear (26). However, metal bearings should be avoided because the metal head wears easily (19) and may even cause periprosthetic metallosis (29). The use of metal-based articulation and grinding of residual ceramic particles on the femoral head prostheses during revision surgery are associated with cobalt toxicity (47, 48). Excessive cobalt levels can lead to systemic prosthetic hip-associated cobalt toxicity (48). Kim et al. reported a case of fatal heart failure caused by cobalt poisoning after successful revision THA (49). To reduce the wear rate, ceramic on polyethylene bearings are a good choice (16). Although the fourth-generation BIOLOX delta ceramic bearing was developed to reduce wear fragments and improve fracture resistance (50), cases of ceramic fragmentation have still been reported with a BIOLOX delta femoral head on a polyethylene liner (23–25). Using the Delta CoC bearing reduced the breakage rate of the ceramic lining to 0.18% (50), which is better than using conventional ceramic, so it can also be considered in revision surgery.

We report two cases of ceramic fragmentation after THA, describing how it occurred and how we managed it during revision surgery. The first patient was diagnosed with ceramic head fragmentation. It was necessary to find a femoral head with the same taper as the femoral neck preoperatively. If the taper is consistent, the new ceramic head is less likely to fracture. It would be better if there were ceramic heads that could prevent fragmentation, such as the pink Aesculap ceramic head that we used here; its surface is coated with a layer of metal so that makes it more resistant to breakage. To the best of our knowledge, we are the first to report the use of this pink Aesculap ceramic head. We feel that this is an ideal revision material. If a ceramic head with a consistent taper cannot be found, the femur will have to be revised as well.

Our second patient was diagnosed with ceramic liner fragmentation. There were several preoperative options. First, a new ceramic liner could be inserted, but none of the existing ceramic liners were suitable, and the original ceramic liner had been discontinued, so this plan was not viable. Second, we could remove the original acetabular cup and replace it with a new acetabular cup and liner. During the operation, the acetabular cup was well fixed to the bone with no signs of loosening. If we forced the acetabular cup out, we would lose too much bone, so this plan was also rejected. The third option, which we ultimately chose, was to preserve the original acetabular cup and then attach a polyethylene liner to it with bone cement. This procedure was quicker and involved less trauma to the patient, but the long-term fixation outcome was uncertain. In addition, some details needed to be considered during the operation, such as removing the screws and roughing the inner side of the original acetabular cup to facilitate the penetration of bone cement. After we placed the acetabular cup, it needed to be held in position with a constant force until the cement set.

We report two cases of ceramic fragmentation after THA, describing the symptoms in detail and sharing our surgical experience. Ceramic fragmentation can occur at any time after primary THA; the main symptoms are pain and noise, although some patients may be asymptomatic. When performing revision surgery, it is essential to formulate an appropriate surgical plan, select appropriate bearings, and correct intraoperative handing of the components. We are the first to report the use of pink Aesculap ceramic head, which we consider to be an ideal material for revision surgery. All orthopedic surgeons should be alert to the occurrence of ceramic fragmentation after primary THA.

## Data Availability

The datasets presented in this study can be found in online repositories. The names of the repository/repositories and accession number(s) can be found in the article/Supplementary Material.
